# Effectiveness of convalescent plasma therapy in a patient with severe COVID-19-associated acute kidney injury 

**DOI:** 10.5414/CNCS110507

**Published:** 2021-05-25

**Authors:** Zachary Z. Brener, Adam Brenner

**Affiliations:** 1 Department of Medicine, Renal Division,; 2 Icahn School of Medicine, and; 3 ICU, Mount Sinai Medical Center, Brooklyn, NY, USA

**Keywords:** COVID-19, acute kidney injury, SARS-CoV-2, convalescent plasma

## Abstract

The outbreak of coronavirus disease 2019 (COVID-19) caused by the severe acute respiratory syndrome coronavirus 2 (SARS-CoV-2) has rapidly evolved into a global pandemic. Recent findings indicate an increased risk for kidney involvement, including progressive acute kidney injury (AKI) during COVID-19 infection, specifically in critically ill patients, and associated with high mortality rates. As no specific treatment options exist for AKI secondary to COVID-19, intensive care is largely supportive with a frequent need for renal replacement therapy (RRT). Convalescent plasma (CP) has been approved as an emergency investigational drug with clinical benefits in observational studies. We described a first case of a patient with severe COVID-19 and AKI who had remarkable improvement in his respiratory status and in kidney function following CP therapy. Our findings demonstrate important therapeutic implications of effective multimodality therapy including CP when treating patients with COVID-19 and AKI.

## Introduction 

Although pulmonary involvement and respiratory failure are the main features of coronavirus disease 2019 (COVID-19) infection, recent findings indicate an increased risk for acute kidney injury (AKI) during the course of COVID-19 infection [1, 2, 3, 4, 5]. AKI is more common among patients with more severe disease, particularly in the intensive care unit (ICU) setting, and is considered a negative prognostic factor with respect to survival [4, 6, 7]. The pathophysiologic mechanisms leading to AKI in COVID-19 infection are not yet fully elucidated but may include the direct cytopathic effect of acute respiratory syndrome coronavirus 2 (SARS-CoV-2) on renal tubular and endothelial cells, and indirectly the effect of virus-induced cytokine release contributing to hypoperfusion-related kidney injury [4, 8, 9]. Care for COVID-19 infection complicated with AKI remains largely supportive with a need for renal replacement therapy (RRT) in up to 20% of patients, with mortality exceeding 65% [4, 10]. Convalescent plasma (CP) therapy might be a promising treatment option for severe COVID-19 cases [11, 12, 13]. To our knowledge, this is the first report of a patient with severe COVID infection and AKI who dramatically improved after CP therapy without the need for RRT. 

## Case report 

An 81-year-old Caucasian man with a history of hypertension, type 2 diabetes mellitus, cardiovascular disease, hyperlipidemia, and benign prostate hypertrophy presented with a fall at home without witnessed syncope or seizures. His medications consisted of clopidogrel, enalapril, finasteride, gabapentin, glimepiride, sitagliptin, metformin, and tamsulosin. 

At presentation at the Mount Sinai Medical Center, Brooklyn, New York, in September 2020, the patient complained of weakness, fatigue, and frequent urinations. His body temperature was 39.0 °C, and blood pressure was 150/95 mm Hg, with a pulse rate of 98 beats/minute and oxygen saturation rate of 98%. He was alert and oriented, with unremarkable lung and cardiovascular examination. Laboratory findings showed leukocytes, 6.6 × 10^3^/µL; hemoglobin, 12.2 g/dL; thrombocytes, 89 × 10^3^/µL; glucose, 235 mg/dL; sodium, 134 mEq/L; potassium 4.6 mEq/L; bicarbonate, 16 mEq/L; calcium, 8.5 mEq/L; serum creatinine (Scr) 1.1 mg/dL; blood urea nitrogen (BUN), 29 mg/dL; creatine phosphokinase (CPK), 9,128 IU/L; prothrombin time, 13.4 s; partial thromboplastin time, 25.3 s. Urinalysis and urinary microscopy demonstrated glucose, erythrocytes, and trace protein and no casts. A chest X-ray showed clear lungs. COVID-19 infection was diagnosed based on a reverse transcriptase-polymerase chain reaction test that detected acute respiratory syndrome coronavirus 2 (SARS-CoV-2) in a throat swab sample. Initial treatment was supportive, with nasal cannula oxygen, doxycycline, and thrombosis prophylaxis with apixaban. 

**Figure 1:**
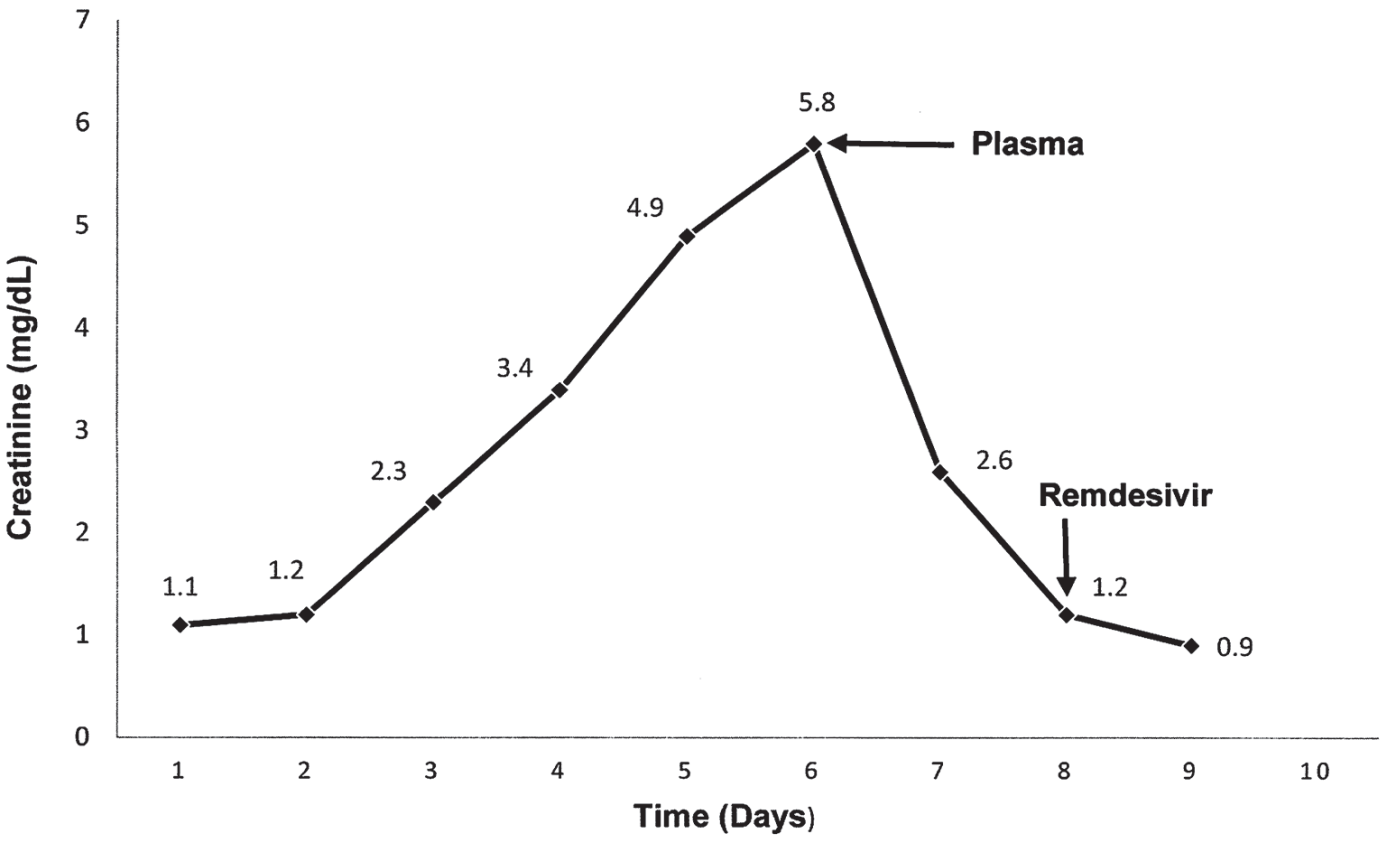
Time course of the serum creatinine over the duration of hospitalization.

**Figure 2:**
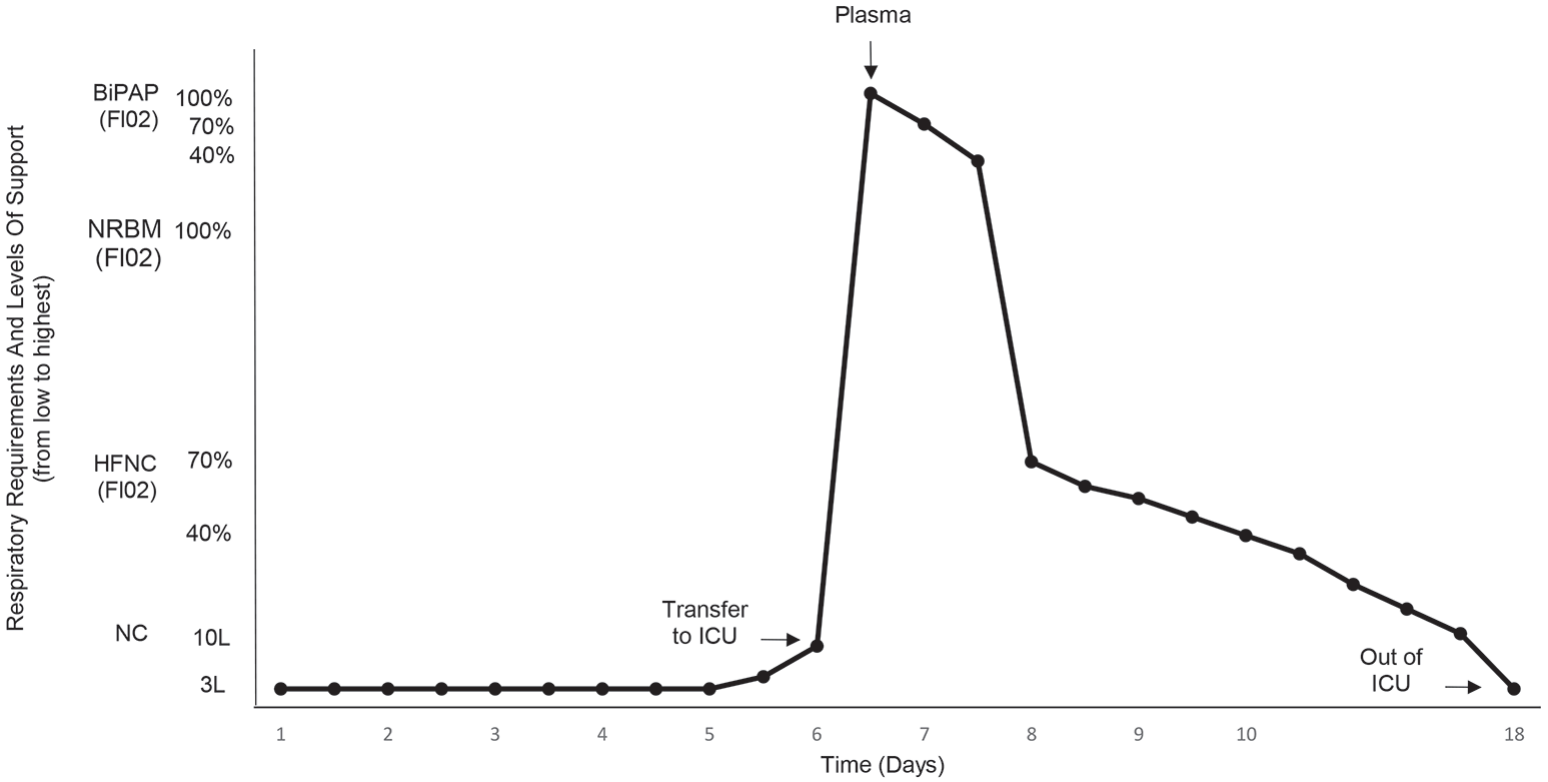
Time course of respiratory requirements over the course of hospitalization.

On day 5 after admission, the condition of the patient worsened. He became more tachypneic, with oxygen saturation decreased to 87% on nasal cannula. There were no episodes of hemodynamic instability. He developed non-oliguric deterioration of kidney function, with Scr and BUN increased to a maximum level of 5.8 mg/dL and 124 mg/dL, respectively. Arterial blood gas on non-rebreather mask showed pH 7.25, Pa O_2_ 78 mmHg, Pa CO_2_ 31 mmHg, and HCO_3_ 13 meq/L. Chest X-ray showed diffuse patchy bilateral infiltrates. The patient was transferred to the Intensive Care Unit (ICU), started BiPAP therapy, intravenous steroids, and was given convalescent plasma. Over the next 48 hours, his respiratory status started improving, with Scr decreasing to 1.2 mg/dL, and CPK to 280 IU/L (Figure 1). He was changed to high-flow oxygen therapy (HFOT) (Figure 2) and given 5 days of remdesivir treatment. 

In the following period, the patient showed gradual respiratory improvement and diuresis. At 20 days after admission, he was discharged from the ICU, and after 43 days, when his throat swab test for coronavirus became negative, he was discharged home. At the time of discharge, Scr level was 0.9 mg/dL. 

## Discussion 

In December 2019, a new strain of coronavirus was identified and named severe acute respiratory syndrome coronavirus 2 (SARS-CoV-2) [14]. Over the past 10 months, COVID-19 has afflicted over 61 million patients and resulted in over 1.4 million deaths. The United States has become the worst-affected country, with more than 300,000 diagnosed cases in New York City and nearly 24,000 virus-related deaths there. 

Acute respiratory distress syndrome due to diffuse alveolar damage has been associated with highest morbidity and mortality, but it is now being described that involvement of the other organs, such as the heart, kidney, and nervous system, may occur and influence the clinical course and patient outcome. Renal involvement with proteinuria, hematuria, and changes in kidney function might be present in up to 75% of cases [1]. AKI is more common among critically ill patients with COVID-19, affecting ~ 20 – 40% of patients admitted to intensive care, according to experience in Europe and the USA, and it is considered a risk factor associated with disease severity and high in-hospital mortality [15]. These patients were significantly older, more likely male, and with other comorbidities, including chronic kidney disease, hypertension, and cardiovascular disease [1]. Compared to patients without AKI, patients with AKI were more likely to receive vasopressors, mechanical ventilatory support, and receive RRT during hospitalization. In our hospital ICU experience in March, April, and May 2020, we found that ~ 80% of critically ill intubated patients developed renal failure, the majority of whom died. In March, only 2 patients who had renal failure requiring renal replacement therapy survived and actually had renal recovery. 

The exact mechanism of kidney involvement is likely to be multifactorial with direct tubular and endothelial damage through an angiotensin-converting enzyme 2 (ACE2)-dependent pathway causing mitochondrial dysfunction, acute tubular necrosis, collapsing glomerulopathy, and protein leakage in Bowman’s capsule [8]. Other indirect mechanisms that potentially lead to tubular injury are sepsis, cytokine release syndrome, hemodynamic instability, rhabdomyolysis, and the development of microemboli and microthrombi in the context of hypercoagulability [10]. 

Early recognition of kidney involvement in COVID-19 and use of preventive and therapeutic measures to limit progression of AKI are crucial to reduce morbidity and mortality. As no specific treatment options exist for AKI secondary to COVID-19, intensive care is largely supportive. Current approaches to prevention and management of AKI, and identification of potential indications for use of RRT are based largely on clinical experience, and AKI strategies are adjusted empirically to patients with COVID-19 [4, 10]. Remdesivir, an adenosine analogue antiviral agent, first developed in response to Ebola, has broad-spectrum antiviral activity and, in a large clinical trial, was shown to bring about faster recovery in patients with COVID-19 than in patients who received placebo [16]. Numerous randomized-controlled trials of this drug are ongoing to determine its safety and efficacy. Moreover, it has not been recommended in patients with estimated glomerular filtration rate (eGFR) of less than 30 mL/min due to the predominately renal excretion of its metabolite (GS-441524) and solubility agent sulfobutylether-beta-cyclodextrin (SBECD). The patient in our case was given remdesivir when his kidney function and clinical condition began improving, thus making effectiveness of antiviral therapy less certain. Therefore, it is an urgent need to look for an alternative strategy for COVID-19 treatment, especially among severe patients with AKI. 

CP therapy, a classic adaptive immunotherapy, has been applied to the prevention and treatment of many bacterial and viral diseases since the 1880s. The use of CP was recommended as an empirical treatment during the outbreak of Ebola virus in 2014 and as a protocol for treatment of sever acute respiratory syndrome (SARS) and Middle East respiratory syndrome (MERS) [17]. On March 24, 2020, the United States Food and Drug Administration (FDA) announced the approval of CP for critically ill patients with COVID-19 as an emergency new investigational drug. Randomized trials have not demonstrated a clear clinical benefit of convalescent plasma [12, 13]. Use of convalescent plasma for severe COVID-19 has also been reported in observational studies, several of which suggest that administration of convalescent plasma with higher antibody titers and earlier in presentation are associated with a greater clinical effect [18]. Initial safety assessment of 5,000 patients who received CP therapy in the USA demonstrated a < 1% rate of serious adverse events immediately following treatment, indicating that risks of CP therapy are likely much smaller than the risks of severe COVID-19 [19]. 

In conclusion, we presented a case of a patient with severe COVID-19 and AKI who had rapid and substantial improvement in his respiratory status and in kidney function following CP therapy. The in-hospital mortality of patients with COVID-19 with AKI is extremely high, and our findings may have a significant impact on therapeutic approach in management of these patients. Therefore, we recommend that physicians carefully monitor kidney function from the early period and administer effective multimodality therapy including CP when treating patients with COVID-19 and AKI. 

## Funding 

There was no support/funding for this report. 

## Conflict of interest 

There is no potential conflict of interest.
